# Radiomics-Based Preoperative Prediction of Lymph Node Status Following Neoadjuvant Therapy in Locally Advanced Rectal Cancer

**DOI:** 10.3389/fonc.2020.00604

**Published:** 2020-05-11

**Authors:** Xuezhi Zhou, Yongju Yi, Zhenyu Liu, Zhiyang Zhou, Bingjia Lai, Kai Sun, Longfei Li, Liyu Huang, Yanqiu Feng, Wuteng Cao, Jie Tian

**Affiliations:** ^1^Engineering Research Center of Molecular and Neuro Imaging of Ministry of Education, School of Life Science and Technology, Xidian University, Xi'an, China; ^2^CAS Key Laboratory of Molecular Imaging, Institute of Automation, Chinese Academy of Science, Beijing, China; ^3^Guangdong Provincial Key Laboratory of Medical Image Processing, School of Biomedical Engineering, Southern Medical University, Guangzhou, China; ^4^Network Information Center, The Sixth Affiliated Hospital, Sun Yat-sen University, Guangzhou, China; ^5^University of Chinese Academy of Science, Beijing, China; ^6^Department of Radiology, The Sixth Affiliated Hospital, Sun Yat-sen University, Guangzhou, China; ^7^Department of Radiology, Sun Yat-sen Memorial Hospital, Sun Yat-sen University, Guangzhou, China; ^8^Collaborative Innovation Center for Internet Healthcare, Zhengzhou University, Zhengzhou, China; ^9^Beijing Advanced Innovation Center for Big Data-Based Precision Medicine, School of Medicine, Beihang University, Beijing, China

**Keywords:** lymph node metastasis, prediction, neoadjuvant therapy, locally advanced rectal cancer, radiomics

## Abstract

**Background and Purpose:** Lymph node status is a key factor for the recommendation of organ preservation for patients with locally advanced rectal cancer (LARC) following neoadjuvant therapy but generally confirmed post-operation. This study aimed to preoperatively predict the lymph node status following neoadjuvant therapy using multiparametric magnetic resonance imaging (MRI)-based radiomic signature.

**Materials and Methods:** A total of 391 patients with LARC who underwent neoadjuvant therapy and TME were included, of which 261 and 130 patients were allocated to the primary cohort and the validation cohort, respectively. The tumor area, as determined by preoperative MRI, underwent radiomics analysis to build a radiomic signature related to lymph node status. Two radiologists reassessed the lymph node status on MRI. The radiomic signature and restaging results were included in a multivariate analysis to build a combined model for predicting the lymph node status. Stratified analyses were performed to test the predictive ability of the combined model in patients with post-therapeutic MRI T1-2 or T3-4 tumors, respectively.

**Results:** The combined model was built in the primary cohort, and predicted lymph node metastasis (LNM+) with an area under the curve of 0.818 and a negative predictive value (NPV) of 93.7% were considered in the validation cohort. Stratified analyses indicated that the combined model could predict LNM+ with a NPV of 100 and 87.8% in the post-therapeutic MRI T1-2 and T3-4 subgroups, respectively.

**Conclusion:** This study reveals the potential of radiomics as a predictor of lymph node status for patients with LARC following neoadjuvant therapy, especially for those with post-therapeutic MRI T1-2 tumors.

## Introduction

Neoadjuvant therapy followed by total mesorectal excision (TME) is the standard treatment for patients with locally advanced rectal cancer (LARC) (1). After neoadjuvant therapy, ~50–60% of patients are downstaged, and ~20% show pathologic complete response (1–3). Although TME is effective at providing local tumor control, it is also associated with significant genitourinary and gastrointestinal morbidity and long-lasting complications such as sexual dysfunction and urinary or fecal problems (4–6). Hence, organ preservation strategies, such as watchful waiting and local excision (7) following neoadjuvant therapy, are becoming more popular for preserving organ function and improving the patients' quality of life (8–12).

One of the disadvantages of organ preservation is a lack of exact pathologic lymph node staging. Leaving lymph node metastasis (LNM+) unresected can potentially lead to local recurrence or distant spread. Magnetic resonance imaging (MRI) and computer tomography are the routine imaging modalities for restaging following neoadjuvant therapy for rectal cancer, but with limited accuracy and with no consensus regarding the standard definitions of LNM+ (13). Neoadjuvant therapy results in changes in shape, size, and texture of a positive lymph node, but these changes still cannot exactly indicate a positive node turning out to be negative. The remains of tumor cells in small nodes make nodal restaging a challenge, which makes patients to have to undergo TME to obtain the precise pathological nodal stage (14). Several studies have investigated the predictive factors for LNM+ but have not identified measures with sufficient predictive precision to enable clinical decisions. For example, a nomogram based on preoperatively available clinicopathologic features has been created to predict LNM+ following neoadjuvant treatment for LARC. If the threshold of 0.3 nomogram predicting the risk of positive nodes is used, almost 80% of the patients with LNM+ will be correctly identified (15). Azizian et al. found that changes of circulating miR-18b and miR-20a expression levels during neoadjuvant treatment could predict LNM+ with a NPV of 79 and 85%, respectively (16). A recent study reported that two factors (ypT stage <3 and lymphovascular invasion) were associated with ypN0 status in good responders following neoadjuvant therapy, indicating a high positive predictive value (PPV) for identifying ypN0 patients (17). However, this study had a small sample size and lacked validation, and the predictive factors were derived from resection specimens; this precluded desirable preoperative decision-making.

Radiomics is a rapid developing field of quantitative image analysis that may facilitate the prediction of lymph node status following neoadjuvant therapy (18, 19). The utility of radiomics is evident from clinical research, such as the prediction of therapeutic responses (20–23), survival analysis (24, 25), and prediction of clinical events (26, 27). Recently, two studies (28, 29) have attempted to detect the associations between local tumor region information on imaging and surrounding nodals and demonstrated the potential of preoperative tumor radiomic features in predicting LNM+ in rectal cancer; however, their analyses were limited to patients that were not administered with any preoperative treatment. Therefore, we hypothesize that local tumor region information following neoadjuvant therapy may also associate with regional nodal status.

Radiomics could quantitatively analyze image information, which may help to detect some associations between local tumor information on imaging and surrounding nodal status. This study aimed to assess if preoperative MRI-based radiomic features could reliably predict lymph node status following neoadjuvant therapy in LARC to improve patient management. Briefly, we first attempted to construct a multiparametric MRI-based radiomic signature. Then, we built and validated a prediction model incorporating the radiomic signature and radiologist's assessment results. Finally, we evaluated the prediction model's performance in two subgroups with different post-therapeutic MRI T (ymrT) stages to identify the ideal population in which this model would be applicable.

## Materials and Methods

### Patients

**Figure 1:**
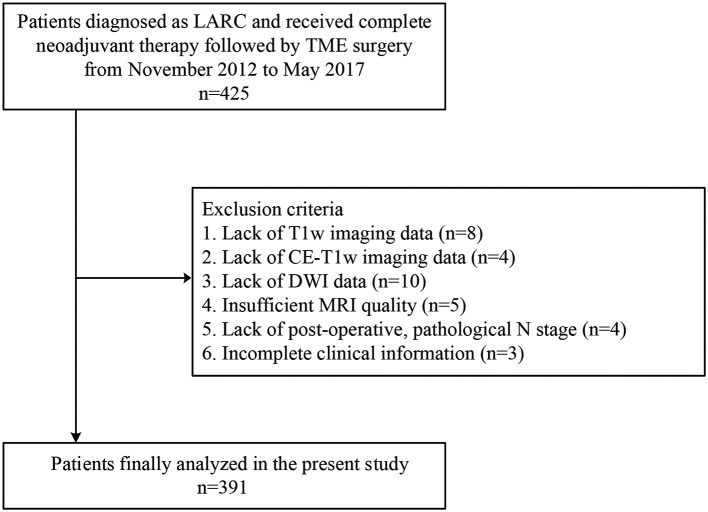
Recruitment pathway for patients in this study. LARC, locally advanced rectal cancer; T1w, T1-weighted; CE-T1w, contrast-enhanced T1-weighted; DWI, diffusion-weighted imaging; MRI, magnetic resonance imaging.

This retrospective study was approved by the institutional review board of the Sixth Affiliated Hospital of Sun Yat-sen University. The requirement for informed patient consent was waived. A total of 425 patients who were initially diagnosed with N+ or T3/T4 rectal cancer, also named as LARC, and received neoadjuvant therapy followed by TME surgery between November 2012 and May 2017 at the Sixth Affiliated Hospital of Sun Yat-sen University were included. The exclusion criteria were as follows: (i) lack of multiparametric MRI data including T1-weighted fast spin-echo imaging (T1w), T2 weighted fast spin-echo imaging (T2w), diffusion-weighted imaging (DWI), or contrast-enhanced T1-weighted fast spin-echo imaging (CE-T1w) 1 week before TME surgery; (ii) insufficient MRI quality due to bowel peristalsis-related artifacts; (iii) lack of clinical information including sex, age, and carcinoembryonic antigen (CEA) (cutoff: ≥ 5 ng/ml, <5 ng/ml) blood level; and (iv) lack of pathology reports, since the pathological lymph nodal status will be obtained from the pathology reports. The recruitment of patients is depicted in Figure 1. Patients were then randomly allocated to a primary cohort and a validation cohort in a ratio of 2:1.

### Multiparametric MRI Acquisition

All patients were scanned with a 1.5-Tesla MR (Optima MR 360, GE Medical Systems, USA) using an eight-element body array coil with fixed image protocols. The scanning sequences consisted of T1w, T2w, DWI (two b-values including 0 and 800 s/mm^2^), and CE-T1w. The technical MRI parameters are listed in Supplementary Table A2.

### Tumor Masking and Radiomic Feature Extraction

**Figure 2:**
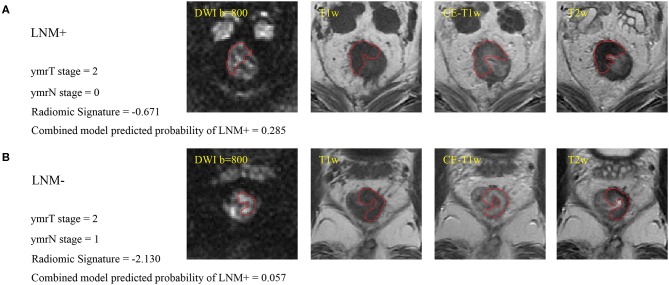
**(A)** Sample images of LNM+, where the red line indicates the tumor margin. **(B)** Sample images of LNM–. Cutoffs for the radiomic signature and combined model are −1.4208 and 0.0897, respectively, for patients with ymrT1-2 tumors in this study. These two patients were misdiagnosed by the radiologist but were correctly assessed by radiomics analysis. LNM+, lymph node metastasis; LNM–, lymph node non-metastasis; DWI, diffusion-weighted imaging; T1w, T1-weighted; CE-T1w, contrast-enhanced T1-weighted; T2w, T2-weighted.

Two gastrointestinal radiologists with 5 (radiologist #1) and 10 (radiologist #2) years of experience examined the MR images and independently defined the regions of interest by manually outlining the tumor margin using itk-SNAP software (www.itksnap.org) on axial slices containing the largest cross-sectional tumor area on each imaging sequence, as shown in Figure 2. At an intuitive level, the most reasonable way to predict the lymph node status is to perform radiomic analysis on each node. However, doing so in this retrospective study is almost impossible as it needs to know every node's pathological status and needs to map every lymph node tissue on MRIs. In this study, we could only obtain the patient-level lymph node status from the post-operative pathology report, which was the number of positive nodes and all nodes from the resection specimens. There even existed some small nodes that could be identified under the microscope but are missed on MRIs following neoadjuvant therapy. In addition, one problem must be solved if we perform radiomic analysis on identifiable nodes on MRIs. The number of identifiable nodes can vary a lot between different patients. That means that we will obtain feature sets with different feature numbers between different patients. Transforming these feature sets into the same feature space is difficult to solve. Thus, we defined a local tumor area as a region of interest like most of the published study (28, 30, 31). Local tumor region information following neoadjuvant therapy may also associate with regional nodal status. Intraclass correlation coefficients were used to assess the agreement of extracted features by two radiologists. The regions of interest on DWI were delineated at a *b* value of 800 s/mm^2^ and were then copied onto the corresponding apparent diffusion coefficient (ADC) maps.

The radiomic features extracted are listed in Supplementary Table A3. A total of 264 features were extracted from each of the T1w, T2w, and CE-T1w images and the ADC maps. These features could be divided into three categories, including first-order statistics, textural features, and Laplacian of Gaussian (LoG) filtration features. Radiomic feature extraction was conducted using an in-house software written in MATLAB (MathWorks, Inc., Natick, MA, USA). All features were linearly normalized into a range [0, 1] with the formula as follows:

(1) $$\begin{array}{l} X_{i}^{norm}=\left(X_{i}-X_{i}^{\min}\right)/\left(X_{i}^{\max}-X_{i}^{\min}\right) \end{array}$$

where $X_{i}^{norm}$ was the *ith* normalized feature value, *X*_*i*_ was the *ith* raw feature value, and $\;X_{i}^{\min}$ and $X_{i}^{\max}$ were the minimum value and maximum value of the *ith* raw feature values in the primary cohort, respectively.

### Feature Selection and Radiomic Signature Construction

We built a model for predicting LNM+ in the primary cohort and evaluated its generalizability in the validation cohort. Before modeling, a feature selection program consisting of three steps was executed in the primary cohort. First, the Wilcoxon rank-sum test was performed for every feature between the LNM+ and LNM- groups as a rough identification of features with *p* ≤ 0.1 to be used in further processing. Second, the Spearman correlation coefficient was calculated between any two features, and the feature with the bigger Wilcoxon rank-sum test *p*-value was excluded when the absolute value of the correlation coefficient exceeded 0.9. Third, the least absolute shrinkage and selection operator (LASSO) method was applied to select the most predictive features (32). To avoid over-fitting, the best LASSO regularization parameter “lambda” was determined by a 10-fold cross-validation. Features with one standard error from the minimum criterion were selected for modeling. Then, a multivariate logistic regression model was built based on the selected features. Summation of the selected features multiplied by the corresponding coefficients was performed for each patient as a radiomic signature, which was mathematically represented as follows:

(2) $$\begin{array}{l} \text{radiomic signature}=\overset{n}{\underset{i=1}{\sum }}C_{i}^{*}X_{i}+b \end{array}$$

(3) $$\begin{array}{l} Y=1/\left(1+\text{exp}\left(-\left(\overset{n}{\underset{i=1}{\sum }}C_{i}^{*}X_{i}+b\right)\right)\right) \end{array}$$

where *Y* was the probability of LNM+ predicted by this model, *b* was the intercept, *X*_*i*_ was the *ith* selected feature, and *C*_*i*_ was the coefficient of the *i*th selected feature. Receiver operating characteristic (ROC) curve analysis was performed in both cohorts to evaluate the predictive ability of radiomic signatures in differentiating LNM+ from LNM-. All steps were performed with R version 3.5.2 (www.r-project.org) using the “glmnet,” “glm2,” and “pROC” packages.

### Comparison of Radiomic Signature and Radiologists' Diagnostic Performance

Radiologists #1 and #2, who were blinded to any medical record information, independently reviewed the MRIs and independently determined the post-therapeutic ymrT stage and post-therapeutic MRI N (ymrN) stage. The ymrT stage was based on the depth of tumor penetration (mucin or soft components) relative to the muscularis propria as T1 (limited to the mucosa and submucosa), T2 (invasion but no penetration of the muscularis propria), T3 (penetration beyond the muscularis propria), or T4 (involvement of other organs). The ymrN status was defined as positive metastasis if the regional lymph node manifested with a small diameter (≥ 6 mm), irregular border, mixed signal intensity (SI), or high SI assumed to represent mucin. The N stage was based on the number of positive lymph nodes: N1 (at least one but less than three nodes) or N2 (more than or equal to three nodes). If the smallest diameter of the largest lymph node was <6 mm and had no features of irregular border and no mixed SI was observed, the N status was graded as N0 (33). Mcnemar test (34) and net reclassification improvement (NRI) test (35) were used for statistical analysis of the prediction results of the radiomic signature and radiologists' diagnosis. Univariate logistic regression analysis was performed in the primary cohort to select the clinical variables with a significant association. Finally, we established a combined model incorporating the radiomic signature and the associated clinical variables by multivariate logistic regression and evaluated this model in the validation cohort. A clinical model incorporating associated clinical variables without radiomic signature was also built through multivariate logistic regression for comparison purposes. To provide an easily used quantitative tool to predict the probability of LNM+, we converted the combined model to a nomogram. The calibration curves were plotted to assess the consistency between the predicted probability and the actual rate of LNM+. Hosmer-Lemeshow test with *p*-value > 0.05 indicates a good fit of the model (36). Decision curve analysis was also conducted to assess the clinical use of this nomogram.

Unlike patients with ymrT3-4 tumors, patients with ymrT1-2 tumors usually exhibit a lower probability of LNM+ and a smaller depth of invasion (37); thus, they are more suitable candidates for local excision. The predictive ability of the model may differ in subgroups divided according to ymrT stage. Thus, we conducted stratified analyses in ymrT1-2 and ymrT3-4 groups, respectively.

Area under the curve (AUC), accuracy, sensitivity, specificity, PPV, and negative predictive value (NPV) according to the Youden cutoff (38) were calculated to quantize the predictive ability of the prediction models in both cohorts.

## Results

### Demographic and Clinical Data

A total of 391 patients were enrolled in the study, as described in Figure 1; 231 of these patients underwent preoperative treatment with four to six cycles of mFOLFOX6 chemotherapy (infusional fluorouracil plus oxaliplatin of 85 mg/m^2^ intravenously on day 1 of each chemotherapy cycle). Postoperative adjuvant chemotherapy was performed with seven cycles of mFOLFOX6; the rest of the 160 patients received preoperative treatment with five cycles of infusional fluorouracil (leucovorin 400 mg/m^2^ intravenously followed by fluorouracil 400 mg/m^2^ intravenously and fluorouracil 2.4 g/m^2^ by 48-h continuous intravenous infusion) and concurrent radiation treatment. Radiotherapy was delivered at 1.8 to 2.0 Gy daily from Monday through Friday for a total of 23 to 28 fractions over 5 to 6 weeks and a total dose of 46.0 to 50.4 Gy. Radiation was delivered with a minimum energy of 6-MV photons through a three- or four-field box technique to the primary tumor and to mesorectal, presacral, and internal iliac lymph nodes (39). A post-operative pathological examination indicated that 87 patients were LNM+. The number of positive nodes ranges from 1 to 12, with a median number of 2. The other 304 patients were LNM-. The clinical characteristics of the patients enrolled are summarized in Table 1 and in Supplementary Table A1. There were no significant differences in the clinical variables between the primary and the validation cohorts. Table 2 exhibited the agreement of ymrT/N stage and ypT/N stage. The ymrT could predict ypT stage with an accuracy of 88.2%. The major predicted error was derived from overstaging of ypT0–2. However, in terms of node restaging, ymrN and ypN showed bad concordance.

**Table 1:** Clinical characteristics of patients in primary and validation cohorts.

**Characteristic**	**Primary cohort (*****n*** **=** **261)**		***p***	**Validation cohort (*****n*** **=** **130)**		***p***
	**LNM+ (*n* = 58)**	**LNM- (*n* = 203)**		**LNM+ (*n* = 29)**	**LNM- (*n* = 101)**	
Age, years	50.24 ± 11.76	54.61 ± 12.69	0.203	53.17 ± 12.95	53.88.06 ± 11.36	0.775
cT stage, *n* (%)			0.520			0.247
T2	2 (4)	14 (7)		0 (0)	9 (9)	
T3	42 (72)	149 (73)		24 (83)	75 (74)	
T4	14 (24)	40 (20)		5 (17)	17 (17)	
cN stage, *n* (%)			**0.003**			0.100
N0	6 (11)	42 (21)		5 (17)	30 (30)	
N1	17 (29)	88 (43)		10 (35)	43 (43)	
N2	35 (60)	73 (36)		14 (48)	28 (27)	
Concurrent radiation, *n* (%)			0.759			0.533
Yes	21 (36)	78 (38)		12 (41)	49 (48)	
No	37 (64)	125 (62)		17 (59)	52 (52)	
Sex, *n* (%)			0.717			0.293
Male	42 (72)	142 (70)		23 (79)	70 (69)	
Female	16 (28)	61 (30)		6 (21)	31 (31)	
CEA, *n* (%)			0.317			0.247
Positive	14 (24)	37 (18)		8 (28)	18 (18)	
Negative	44 (76)	166 (82)		21 (72)	83 (82)	
ymrT stage, *n* (%)			**0.018**			**0.032**
T1	1 (2)	25 (12)		0 (0)	7 (7)	
T2	11 (19)	55 (27)		3 (10)	32 (32)	
T3	36 (62)	105 (52)		23 (80)	57 (56)	
T4	10 (17)	18 (9)		3 (10)	5 (5)	
ymrN stage, *n* (%)			**<0.001**			**0.006**
N0	24 (41)	142 (70)		14 (49)	73 (72)	
N1	19 (33)	51 (25)		10 (34)	25 (25)	
N2	15 (26)	10 (5)		5 (17)	3 (3)	

*Age is presented as mean ± standard deviation. The p-value for age was calculated using independent samples t-test analysis. The p-values for the categorical variables were calculated using Pearson's chi-square test analysis. ymrT stage and ymrN stage were restaged by radiologist #2 who has 10 years of experience. Bold font indicates p < 0.05. LNM+, lymph node metastasis; LNM–, lymph node non-metastasis; CEA, carcinoembryonic antigen; ymr, restaging MRI assessments*.

**Table 2:** Confusion matrice for tumor restaging and node restaging.

	**ypT0-2**	**ypT3-4**		**yN0**	**yN+**
ymrT1–2	120	14	ymrN0	215	38
ymrT3–4	32	225	ymrN+	89	49

*The ymrT/N stages were assessed by radiologist #2 who has 10 years of experience*.

### Radiomic Signature Construction

**Figure 3:**
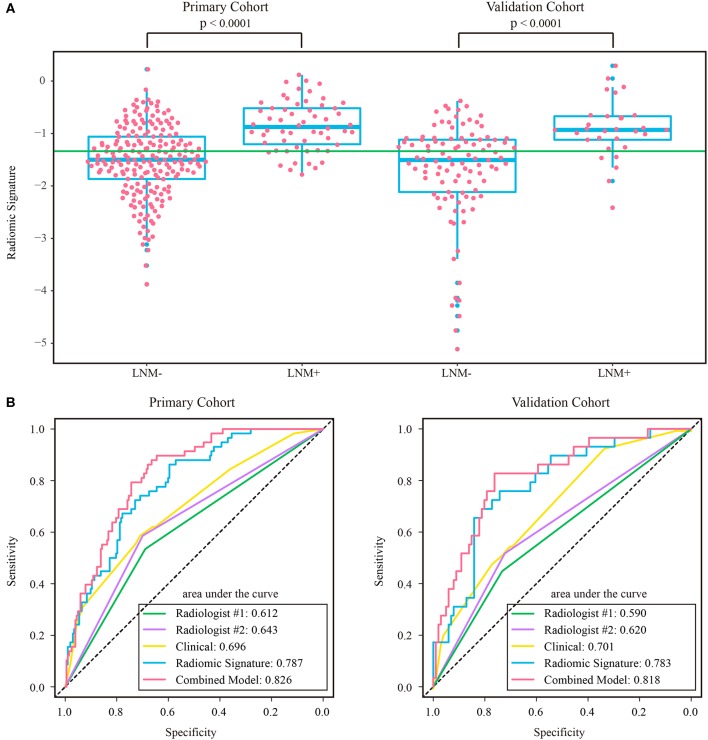
**(A)** Distribution of radiomic signature in the primary and validation cohorts, where the green line indicates the Youden cutoff in the primary cohort, and the *p*-value was calculated using Wilcoxon rank-sum test. **(B)** ROC curves of radiologists' and prediction models. LNM+, lymph node metastasis; LNM-, lymph node non-metastasis; ROC, receiver operating characteristic.

The intraclass correlation coefficients calculated for features extracted by the two radiologists ranged from 0.725 to 0.942, reflecting a good agreement. The features extracted from the regions of interest delineated by the radiologist with 10 years of experience were used for further analysis. Thirteen features were selected to build a radiomic signature, as listed in Supplementary Table A4. None of the T1w feature was selected, indicating a poor predictive ability of T1w features. In both cohorts, the radiomic signature was significantly higher in the LNM+ group than in the LNM- group, as shown in Figure 3. The radiomic signature yielded an AUC of 0.787 [95% confidence interval (CI): 0.726–0.848] and 0.783 (95% CI: 0.690–0.875) in the primary and validation cohorts, respectively.

### Comparison of Radiomic Signature and Radiologists' Diagnostic Performance

The assessment results of the two radiologists were highly consistent, yielding a Kappa value of 0.936 and 0.933 for ymrT stage and ymrN stage, respectively. The confusion matrices, as shown in Supplementary Figure A1, indicated that radiologist #1 and radiologist #2 yielded a sensitivity of 50.57% (95% CI: 35.6–64.7%) and 56.32% (95% CI: 42.3–70.8%), respectively. The AUC of radiologist #2 was 0.62 (95% CI: 0.518–0.722) in the validation cohort, which was significantly (Delong test *p*-value: 0.021) smaller than that of the radiomic signature. The sensitivity of the radiomic signature reached a score of 82.8% (95% CI: 68.8–96.6%), which was significantly (Mcnemar test *p*-value: 0.022) different from that of radiologist #2 in the validation cohort. The specificity values of radiomic signature and radiologist #2 were 58.4% (95% CI: 48.8–67.7%) vs. 72.2% (95% CI: 63.5–81.0%), which were also significantly different (Mcnemar test *p*-value: 0.044).

**Figure 4:**
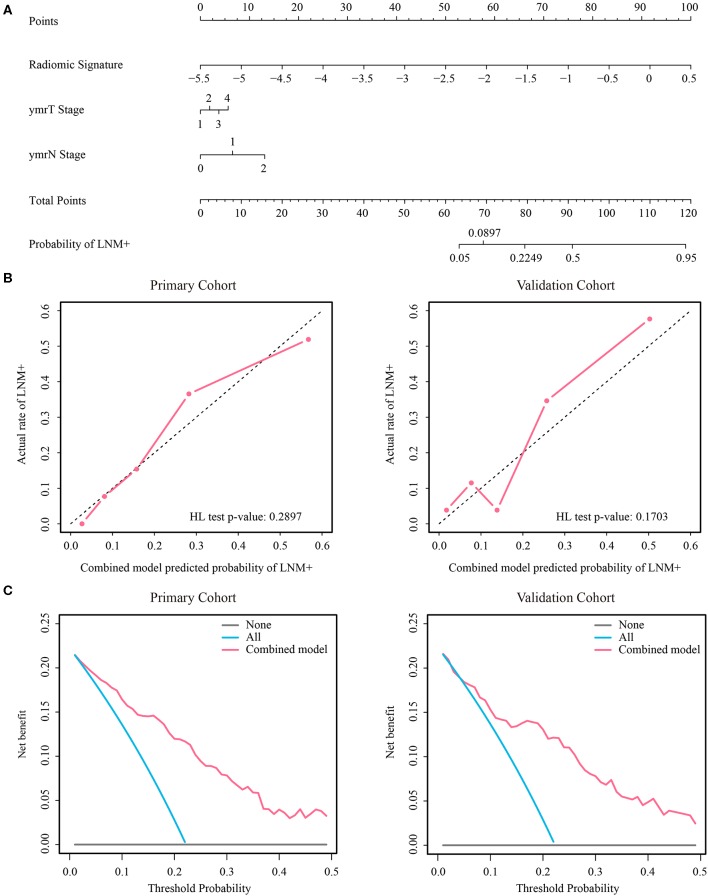
**(A)** Nomogram of the combined model. **(B)** Calibration curves of the combined model in both cohorts. **(C)** Decision curve analysis of the combined model in both cohorts. LNM+, lymph node metastasis; HL, Hosmer-Lemeshow.

As the diagnostic accuracy of radiologist #2 was higher than that of radiologist #1, here we only reported the prediction results based on post-therapeutic restaging results from radiologist #2, and those based on the restaging results from radiologist #1 were provided in the Supplementary File. In univariate logistic regression analysis in the primary cohort, post-therapeutic ymrT stage, ymrN stage, and radiomic signature were statistically significant (Table 3). We built a combined model to integrate the staging results of radiologist #2 and the radiomic signature using multivariate logistic regression in the primary cohort and converted it into a nomogram, as shown in Figure 4. Compared to the radiologists' performance, the prediction accuracy in the validation cohort using the combined model was improved (NRI test *p*-value: 0.125) to 75.4% from the accuracy value of 63.8% of radiologist #2, yielding a sensitivity of 82.8% (95% CI: 68.5–82.8%), specificity of 73.3% (95% CI: 64.8–81.9%), PPV of 47.1% (33.2–60.8%), and NPV of 93.7% (88.3–99.0%). The clinical model incorporating ymrT and ymrN yielded an AUC value of 0.696 (95% CI: 0.619–0.773) and 0.701 (95% CI: 0.601–0.801) in the primary cohort and the validation cohort, respectively. The Delong test analysis showed that the clinical model performed significantly (*p* < 0.05) worse than the combined model but was comparable to the radiomic signature (*p* > 0.05) in both cohorts. All these results are listed in Supplementary Table A5 and Figure 2A.

**Table 3:** Univariate and multivariate logistic regression analysis for clinical characteristics and radiomic signature.

**Parameter**	**Univariate**			**Multivariate**			
	***p***	**OR**	**95% CI**	***p***	**Coefficient**	**OR**	**95% CI**
Sex	0.7169	0.89	0.46–1.69	–	–	–	–
Age	0.2029	0.78	0.53–1.14	–	–	–	–
CEA	0.3183	1.43	0.71–2.87	–	–	–	–
Concurrent radiation	0.7590	0.91	0.49–1.66	–	–	–	–
ymrT stage	**0.0020**	1.93	1.27–2.93	0.4210	0.2000	1.22	0.75–1.99
ymrN stage	**<0.0001**	7.72	3.31–18.02	**0.0063**	0.6963	4.03	1.48–10.94
Radiomic signature	**<0.0001**	6.31	3.45–11.55	**<0.0001**	1.7705	5.15	2.78–9.55
Intercept	–	–	–	0.8685	−0.1320	–	–

*The p-values were calculated using Wald test analysis. Bold font indicates p < 0.05. OR, odds ratio; CI, confidence interval; CEA, carcinoembryonic antigen; ymr, restaging MRI assessments*.

**Figure 5:**
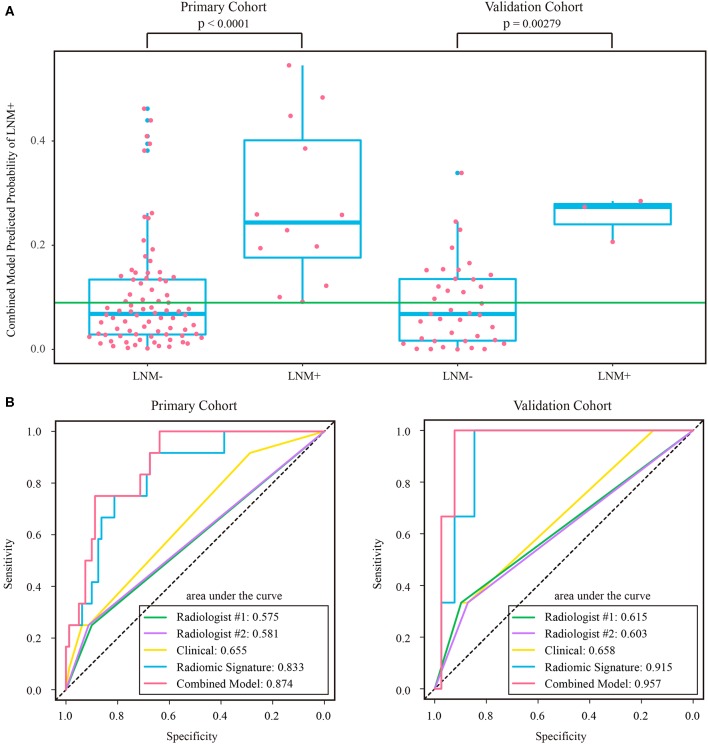
**(A)** Distribution of combined model predicted probability of LNM+ in post-therapeutic ymrT1-2 subgroups of both cohorts, where the green line indicates the Youden cutoff in the primary cohort. **(B)** ROC curves of radiologists' and prediction models in post-therapeutic ymrT1-2 subgroups of both cohorts. LNM+, lymph node metastasis; LNM-, lymph node non-metastasis; ROC, receiver operating characteristic.

**Figure 6:**
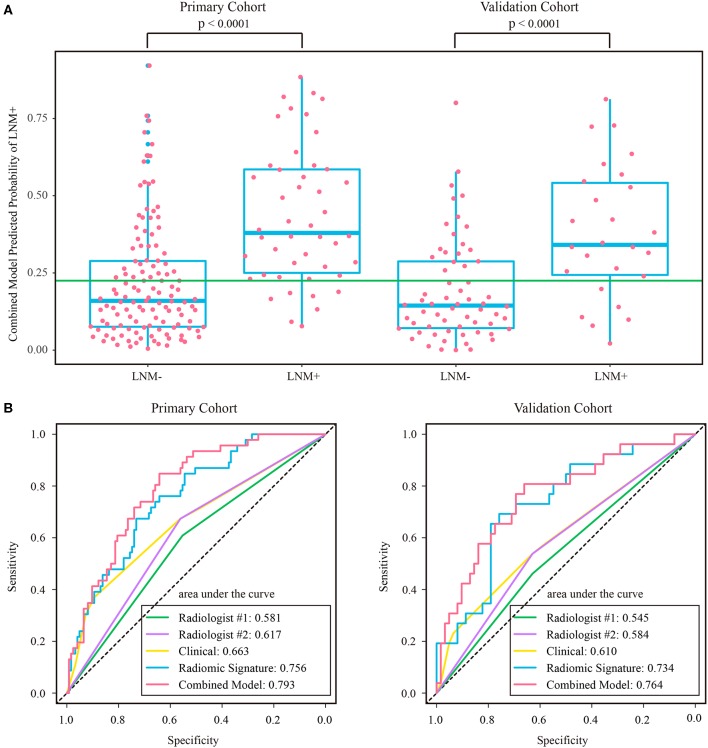
**(A)** Distribution of combined model predicted probability of LNM+ in post-therapeutic ymrT3-4 subgroups of both cohorts, where the green line indicates the Youden cutoff in the primary cohort. **(B)** ROC curves of radiologists and prediction models in post-therapeutic ymrT3-4 subgroups of both cohorts. LNM+, lymph node metastasis; LNM–, lymph node non-metastasis; ROC, receiver operating characteristic.

The stratified analyses indicated that radiologist #2 yielded a better prediction in the ymrT1-2 subgroup than that in the ymrT3-4 subgroup with NPV of 90.7 vs. 80%. The combined model also performed better in the ymrT1-2 subgroup of the validation cohort with an AUC of 0.915 and a NPV of 100%. In the ymrT3-4 subgroup of the validation cohort, the combined model yielded an AUC of 0.764 and a NPV of 87.8% according to the Youden cutoff. Detailed results are shown in Figures 5, 6 and in Supplementary Tables A6, A7. For comparison, the combined model based on radiomic signature and restaging results from radiologist #1 yielded a NPV of 100 and 86.7% in ymrT1–2 subgroup and ymrT3-4 subgroup, respectively (Supplementary Table A8).

## Discussion

In this study, a major finding was that radiomics is a promising approach for the preoperative prediction of LNM+ following neoadjuvant therapy in patients with LARC. Radiomic signature was a powerful predictor independent of the radiologists' diagnostic results, offering a NPV of 92.2% in the validation cohort. Combining radiomic signature with the radiologists' diagnostic results improved the NPV to 93.7%. In the post-therapeutic ymrT1-2 subgroup, the combined model yielded a NPV of 100% and specificity of 59%. However, in the post-therapeutic ymrT3-4 subgroup, the combined model did not achieve 100% NPV.

A major factor limiting the clinical application of organ preservation strategies is that the precise assessment of lymph node status is challenging (14, 40) since the completeness of tumor resection can be determined by pathological examination, but residual LNM+ has a high risk of leading to an adverse prognosis. Although the size and the morphological features (i.e., round shape, irregular border, and heterogeneous texture) have been proposed to define a clinically positive lymph node on MRI, the correspondence between post-therapeutic cN+ and pN+ is still poor. Recently, a large retrospective study from the Netherlands revealed that using post-therapeutic cN+ to predict pN+ yielded a sensitivity of 56%, specificity of 67%, PPV of 47%, and NPV of 75% for rectal cancer patients who received a short course of radiotherapy with short interval to surgery between 2011 and 2014 (41). Our study obtained similar results, whereby the more experienced radiologist's visual assessments could only accurately detect a small proportion of LNM+ with a sensitivity of 56.3%, specificity of 70.7%, PPV of 35.5%, and NPV of 84.9%.

Although receiver operating characteristic analysis indicated that the radiomic signature had superior predictive ability to that of the more experienced radiologist, the radiologist's assessment results should not be overlooked. Compared with the radiomic signature, the radiologist exhibited a lower sensitivity and a higher specificity. In the univariate logistic regression analysis, ymrT and ymrN stages were significantly associated with LNM+. In particular, ymrN stage was still an independent predictor for LNM+ even when considering radiomic signature and ymrT stage as covariates. Thus, based on the advantages of radiomic signature and the radiologist's restaging results, the combined model was able to achieve a higher prediction accuracy.

The results of the stratified analyses highlight the potential of the combined model for clinical application. For patients with post-therapeutic ymrT1-2 tumors following neoadjuvant therapy, the combined model achieved a NPV of 100% and corresponding specificity of 63.8 and 59% in the primary cohort and validation cohort, respectively. This result indicates that approximately 60% of ymrT1-2 patients with LNM- would benefit from the model's prediction results. In practice, choosing less invasive treatment after neoadjuvant therapy for rectal cancer is a difficult and complex decision for both the doctor and the patient. Local excision or wait-and-watch is typically only considered for ypT0-2, lymph node-negative patients. However, we cannot obtain the ypT stage and lymph node status other than by pathologic evaluation after TME. This contradiction spurs us on to achieve a more precise clinical T/N staging. We believe that our combined model can serve as an important assistive tool for assessing the likelihood of node status following neoadjuvant therapy. Further research aiming at the simultaneous precise prediction of ypT stage and ypN before TME is indispensable to promote organ preservation strategies in the clinic.

Radiomics is a data-driven approach which has been successfully used to assess treatment response after neoadjuvant therapy (30) and to predict pathological features such as degree of differentiation, T stage, and N stage (31). It is an advanced framework which selects the most useful features from a high-throughput feature set to build a signature correlated to an object in a linear or a non-linear way. To the best of our knowledge, our study may be one of the first attempts to cope with this clinical problem by using radiomics. The selected radiomic features included understandable first-order statistics features such as LoG3-FOS_Mean, LoG2-FOS_Skewness, and so on, which reflect the strength information of tumor. The selected features also included textural features such as LoG3-GLCM_cshade, LoG2-GLSZM_LZLGE, and so on, which reflect a high-order statistical property among image elements and usually cannot be visually examined, but we believe that these features can be associated with an underlying pathology. Some published studies have mapped radiomic features to gene mutation (42, 43) and molecular pathway activation (44–46) by a radiogenomic method (47, 48). In the future, interpreting these selected features by specific genetic profiles may help to improve decision making in node restaging.

Several limitations existed in this study. It was a retrospective study with single-center samples in China. The chemoradiotherapy regimens usually are not the same in different hospitals, which may cause different lymph node responses. The imaging equipment parameters are usually different in multicenter research, which makes the reliability of the extracted features challenged. In order to control for confounders as much as possible, we conducted our study in a single hospital. Another limitation is that the enrolled sample size was relatively small, especially for the post-therapeutic ymrT1-2 subgroup. Thus, a prospective, international, multicenter clinical trial with a large sample size is needed to confirm our findings. In addition, only two radiologists were involved in the diagnosis in our study, and the more experienced radiologist provided a more accurate diagnosis. Thus, future research should include more experienced radiologists. Perirectal environment is another area that is worth to analyze, but blood vessels, muscles, nerves, and posttreatment edema may exist in this area. These confounding factors may affect the extracted features, causing negative effects to the accuracy of the prediction results. Although manually excluding these confounding factors on MRI is very time-consuming, it is worth trying to analyze the perirectal environment in a further study to get better prediction accuracy. Deep learning is an emerging field that surpasses radiomics in many tasks. Modeling with deep learning to correctly identify more LNM- patients may be a promising direction.

In summary, we demonstrated that combining a radiologist's staging results and radiomics analysis assists in the prediction of lymph node status in patients with LARC following neoadjuvant therapy, especially for patients with post-therapeutic ymrT1-2 tumors. An external validation of this study is warranted to guide the treatment recommendations for patients eligible for organ preservation strategies.

## Data Availability Statement

The datasets for this article are not publicly available as it is private data that belongs to the Sixth Affiliated Hospital of Sun Yat-sen University. Requests to access the datasets should be directed to corresponding author.

## Ethics Statement

The studies involving human participants were reviewed and approved by institutional review board of Sixth Affiliated Hospital of Sun Yat-sen University. The ethics committee waived the requirement of written informed consent for participation.

## Author Contributions

JT, YF, and WC conceptualized and designed the study. Data were acquired by WC, YY, ZZ, and YF. XZ, YY, WC, BL, LL, KS, and LH analyze and interpreted the data. XZ, YY, ZL, and ZZ drafted the manuscript. JT, YF, and WC critically revised the paper.

## Conflict of Interest

The authors declare that the research was conducted in the absence of any commercial or financial relationships that could be construed as a potential conflict of interest.

## Abbreviations

ADC: apparent diffusion coefficient
AUC: area under curve
CI: confidence interval
LARC: locally advanced rectal cancer
LNM+: lymph node metastasis
LNM-: lymph node non-metastasis
LoG: Laplacian of Gaussian
MRI: magnetic resonance imaging
NPV: negative predictive value
PPV: positive predictive value
ROC: receiver operating characteristic
TME: total mesorectal excision
