# Supplementary material for: Radiomics-Based Preoperative Prediction of Lymph Node Status Following Neoadjuvant Therapy in Locally Advanced Rectal Cancer
Source: Front Oncol. 2020 May 11;10:604. doi: 10.3389/fonc.2020.00604 (PMC7233118; doi:10.3389/fonc.2020.00604)
Supplement: Supplementary file 1 [file Data_Sheet_1.docx]

**Supplementary Appendix**

***Radiomics-Based Preoperative*** ***Prediction of*** ***Lymph Node Status Following Neoadjuvant Therapy in Locally Advanced Rectal Cancer***

**Radiomic Feature Extraction**

The radiomic features extracted are listed in **Table A3**. In total, 264 features were extracted from each of T1w, T2w, CE-T1w images, and ADC maps. These features could be divided into 3 categories, including first-order statistics, textural features, and Laplacian of Gaussian (LoG) filtration features. We selected three laplacian of gaussian (LoG) filters for image preprocessing in addition to extracting features from the original images, because LoG filters could concurrently smooth images and enhance textural details, thereby assisting in extraction of features reflecting biological properties of tumors associated with lymph node metastasis. Similar image preprocess was implemented in a CT-based radiomics study for lymph node metastasis prediction (Huang et al., 2016) in which the authors constructed a radiomics signature with 24 features, of which 22 were LoG features. A total of 1056 features were extracted from each patient’s MR images and were linearly normalized into a range [0, 1]. The radiomic feature extraction was conducted using in-house software written in MATLAB (MathWorks, Inc., Natick, MA, USA). All specific calculation formulas could be easily obtained in previous studies (Liu et al., 2017; Yang et al., 2019), thus, here we only provide the names of features we extracted as follows:

(1) **Image filtration**

A Laplacian of Gaussian (LoG) spatial band-pass filter was used to derive image features at different spatial scales by turning the filter parameter among 1.0, 2.0 and 3.0. The LoG filter distribution is given by

$\text{LoG}\left(\text{x},\text{y}\right)=\frac{-1}{\pi\sigma^{4}}\left(1-\frac{x^{2}+y^{2}}{2\sigma^{2}}\right)e^{-\frac{x^{2}+y^{2}}{2\sigma^{2}}}$,

of which $x and y$denote the spatial coordinates of the pixel and $\sigma$is the value of filter parameter.

(2) **First-order statistic (FOS) features**

1. ***Intensity-Max (FOS_Max)***
2. ***Intensity-Min (FOS_Min)***
3. ***Intensity-Median (FOS_Median)***
4. ***Intensity-Mean (FOS_Mean)***
5. ***Intensity-Range (FOS_Range)***
6. ***Intensity-Mean absolute deviation (FOS_MAD)***
7. ***Intensity-Root mean square (FOS_RMS):***
8. ***Intensity-Energy (FOS_Energy)***
9. ***Histogram-Variance (FOS_Variance)***
10. ***Histogram-Skewness (FOS_Skewness)***
11. ***Histogram-Kurtosis (FOS_Kurtosis)***
12. ***Histogram-Entropy (FOS_Entropy)***
13. ***Histogram-*** ***Standard deviation (FOS_Std)***

(3) **Gray-Level Co-Occurrence Matrix (GLCM) based features**

GLCM based features were second-order statistical texture features, which are defined as a matrix $M(i,j;\delta,\theta)$to indicate the relative frequency with intensity values of pixels (i and j) at the distance of$\delta$in direction $\theta$. We set $\delta=1$ and $\theta=0^{\circ}, 45^{\circ},90^{\circ},135^{\circ}$. The number of discrete intensity levels in the images was set as 64. Each 2D GLCM based feature was then calculated as the mean of the feature calculations for each of the 4 directions

1. ***Energy (GLCM_energy)***
2. ***Contrast (GLCM_contrast)***
3. ***Entropy (GLCM_entropy)***
4. ***Homogeneity 1 (GLCM_homogeneity1)***
5. ***Homogeneity 2 (GLCM_homogeneity2)***
6. ***Correlation 1 (GLCM_correlation1)***
7. ***Correlation 2 (GLCM_correlation2)***
8. ***Sum of Squares: Variance (GLCM_variance)***
9. ***Sum Average (GLCM_saverage)***
10. ***Sum Entropy (GLCM_sentropy)***
11. ***Dissimilarity (GLCM_dissimilarity)***
12. ***Autocorrelation (GLCM_autocorrelation)***
13. ***Cluster Prominence (GLCM_cprominence)***
14. ***Cluster Shade (GLCM_cshade)***
15. ***Difference Entropy (GLCM_dentropy)***
16. ***Difference Variance (GLCM_dvariance)***
17. ***Maximum Probability (GLCM_mprobability)***
18. ***Sum variance (GLCM_svariance)***
19. ***Informational measure of correlation 1 (GLCM_IMC1)***
20. ***Informational measure of correlation 2 (GLCM_IMC2)***
21. ***Inverse Difference Moment Normalized (GLCM_IDMN)***
22. ***Inverse Difference Normalized (GLCM_IDN)***

(4) **Gray-Level Run Length Matrix based features (GLRLM)**

GLRLM based features were high-order statistical texture feature, which were defined as $P\left( i,j;\theta\right)$ to indicate the number of times j and gray level i appear consecutively in the direction $\theta$. We set $\theta=0^{\circ}, 45^{\circ},90^{\circ},135^{\circ}$. The number of discrete gray levels in the images was set as 64. A GLRLM was computed for every of the 4 directions in two dimensions, from which the below textural features were derived. Each 2D GLRLM feature was then calculated as the mean of the feature values for each of the 4 directions.

1. ***Short Run Emphasis (GLRLM_SRE)***
2. ***Long Run Emphasis (GLRLM_LRE)***
3. ***Gray-Level Nonuniformity (GLRLM_GLN)***
4. ***Run-Length Nonuniformity (GLRLM_RLN)***
5. ***Gray-Level Variance (GLRLM_GLV)***
6. ***Run-Length Variance (GLRLM_RLV)***
7. ***Run Percentage (GLRLM_RP)***
8. ***Low Gray-Level Run Emphasis (GLRLM_LGRE)***
9. ***High Gray-Level Run Emphasis (GLRLM_HGRE)***
10. ***Short Run Low Gray-Level Emphasis (GLRLM_SRLGE)***
11. ***Short Run High Gray-Level Emphasis (GLRLM_SRHGE)***
12. ***Long Run Low Gray-Level Emphasis (GLRLM_LRLGE)***
13. ***Long Run High Gray-Level Emphasis (GLRLM_LRHGE)***

(5) **Gray Level Size Zone Matrix based features (GLSZM)**

GLSZM based features were high-order statistical texture features, which were defined as $P(i,j)$ to indicate the areas of size j and gray level i. The number of discrete gray levels in the images was set as 64.

1. ***Small Zone Emphasis (GLSZM_SZE)***
2. ***Large Zone Emphasis (GLSZM_LZE)***
3. ***Gray-Level Nonuniformity (GLSZM_GLN)***
4. ***Zone-Size Nonuniformity (GLSZM_ZSN)***
5. ***Gray-Level Variance (GLSZM_GLV)***
6. ***Zone-Size Variance (GLSZM_ZSV)***
7. ***Zone Percentage (GLSZM_ZP)***
8. ***Low Gray-Level Zone Emphasis (GLSZM_LGZE)***
9. ***High Gray-Level Zone Emphasis (GLSZM_HGZE)***
10. ***Small Zone Low Gray-Level Emphasis (GLSZM_SZLGE)***
11. ***Small Zone High Gray-Level Emphasis (GLSZM_SZHGE)***
12. ***Large Zone Low Gray-Level Emphasis (GLSZM_LZLGE)***
13. ***Large Zone High Gray-Level Emphasis (GLSZM_LZHGE)***

(6) **Neighborhood Gray Tone Difference Matrix based features (NGTDM)**

NGTDM based features were high-order statistical texture features, which were defined as $S\left( i \right)$to indicate the sum of the absolute value between gray intensity level i and its neighbors’ average intensity. The number of discrete gray levels in the images was set as 64.

1. ***Coarseness (NGTDM_Coarseness)***
2. ***Contrast (NGTDM_Contrast)***
3. ***Busyness (NGTDM_Busyness)***
4. ***Complexity (NGTDM_Complexity)***
5. ***Strength (NGTDM_Strength)***

**Table A1 Clinical characteristics of patients in the primary and validation cohorts.**

| Characteristic | Primary cohort (n=261) | Validation cohort (n=130) | *p* |
| --- | --- | --- | --- |
| LN status, n (%)  LNM+  LNM- |  |  | 0.985 |
|  | 58 (22) | 29 (22) |  |
|  | 203 (78) | 101 (78) |  |
| Age, years | 54.09 ± 12.51 | 53.72 ± 11.69 | 0.781 |
| Sex, n (%) |  |  | 0.831 |
| Male | 184 (71) | 93 (72) |  |
| Female | 77 (29) | 37 (28) |  |
| CEA, n (%) |  |  | 0.914 |
| Positive | 51 (20) | 26 (20) |  |
| Negative | 210 (80) | 104 (80) |  |
| ymrT stage, n (%) |  |  | 0.166 |
| T1  T2 | 26 (10)  66 (25) | 7 (5)  35 (27) |  |
| T3 | 141 (54) | 80 (62) |  |
| T4 | 28 (11) | 8 (6) |  |
| ymrN stage, n (%) |  |  | 0.508 |
| N0 | 166 (64) | 87 (67) |  |
| N1 | 70 (26) | 35 (27) |  |
| N2 | 25 (10) | 8 (6) |  |

Age is presented as mean ± standard deviation. The p-value for Age was calculated using independent samples t-test analysis. The p-values for categorical variables were calculated using Pearson’s chi-square test analysis. ymrT stage and ymrN stage were restaged by radiologist #2 who has 10 years of experience. LN, lymph node; LNM+, lymph node metastasis; LNM-, lymph node nonmetastasis; CEA, carcinoembryonic antigen; ymr, restaging MRI assessments.

**Table A2 The technical MRI parameters of the scanning sequences.**

| Scanner | Sequence | b value (s/mm^2^) | TR  (ms) | TE  (ms) | Flip Angle | Matrix | Pixel Spacing (mm^2^) | Slice Thickness (mm) | Slice Gap (mm) |
| --- | --- | --- | --- | --- | --- | --- | --- | --- | --- |
| GE 1.5T  (Optima MR360) | DWI | 0/800 | 4600 | 85 | 90^。^ | 256 × 256 | 1.56 × 1.56 | 5 | 6 |
|  | T1w | - | 680 | 13 | 90^。^ | 512 × 512 | 0.55 × 0.55 | 5 | 6 |
|  | CE-T1w | - | 680 | 13 | 90^。^ | 512 × 512 | 0.55 × 0.55 | 5 | 6 |
|  | T2w | - | 4300 | 120 | 90^。^ | 512 × 512 | 0.55 × 0.55 | 5 | 6 |

TR, repetition time; TE, echo time; DWI, diffusion-weighted imaging; T1w, T1-weighted; CE-T1w, contrast-enhanced T1-weighted; T2w, T2-weighted.

**Table A3 Summary of radiomic features extracted in the present study.**

| Original features | LoG filter features |
| --- | --- |
| FOS_name | LoG_σ-FOS_name |
| GLCM_name | LoG_σ-GLCM_name |
| GLRLM_name | LoG_σ-GLRLM_name |
| GLSZM_name | LoG_σ-GLSZM_name |
| NGTDM_name | LoG_σ-NGTDM_name |

σ represents the filter value applied, which could be 1.0, 2.0 and 3.0.

FOS, first-order statistic; LoG, Laplacian of Gaussian; GLCM, gray-level co-occurrence matrix; GLRLM, gray-level run length matrix; GLSZM, gray level size zone matrix; NGTDM, neighborhood gray tone difference matrix.

**Table A4 Risk factors for LNM+ after** **neoadjuvant therapy in LARC.**

| **Intercept and Variable** | **Modality** | **Radiomic Model** | | | | |
| --- | --- | --- | --- | --- | --- | --- |
|  |  | **Minimum value** | **Maximum value** | **Coefficient** | **OR (95% CI)** | ***p*** |
| Intercept | - |  |  | -5.5645 | - | 0.0501 |
| LoG1-FOS_Median | T2w | -19.7362 | 21.2883 | -2.6040 | 0.79 (0.53-1.20) | 0.2807 |
| GLCM_mprobability | T2w | 0.00649 | 0.14035 | 0.4856 | 1.04 (0.43-2.49) | 0.9224 |
| LoG3-FOS_Mean | CE-T1w | -2.06700 | 0.41982 | 7.6516 | 2.15 (1.26-3.67) | 0.0052 |
| GLRLM_GLN | T2w | 0.01985 | 0.10250 | -1.6746 | 0.69 (0.27-1.76) | 0.4376 |
| LoG2-GLSZM_LZLGE | T2w | 0.00198 | 0.06031 | -7.5782 | 0.58 (0.32-1.07) | 0.0796 |
| FOS_Min | T2w | -22.8303 | 19.7142 | 3.2699 | 1.65 (1.08-2.52) | 0.0200 |
| FOS_Range | T2w | 70.7934 | 522.4276 | -0.0968 | 0.97 (0.49-1.94) | 0.9384 |
| LoG1-GLCM_energy | CE-T1w | 0.00305 | 0.03196 | -2.4357 | 0.76 (0.45-1.28) | 0.3024 |
| LoG1-FOS_Max | ADC | 0.000222 | 0.000835 | -5.0445 | 0.49 (0.31-0.79) | 0.0040 |
| LoG2-FOS_Skewness | CE-T1w | -4.73298 | 0.00371 | -1.2841 | 0.79 (0.50-1.25) | 0.3208 |
| LoG3-GLCM_cshade | ADC | -49169.77 | 5924.56 | 3.5817 | 2.03 (1.29-3.19) | 0.0024 |
| GLCM_variance | ADC | 29.5301 | 664.4519 | -1.1213 | 0.74 (0.46-1.19) | 0.2215 |
| LoG1-FOS_Skewness | T2w | -3.25774 | 1.88558 | 2.0311 | 1.53 (0.93-2.50) | 0.0916 |

The p value was from Wald test analysis.

OR, odds ratio; LoG, Laplacian of Gaussian; FOS, first-order statistic; GLCM, gray-level co-occurrence matrix; GLRLM, gray-level run-length matrix; GLSZM, gray-level size zone matrix; T2w, T2-weighted; CE-T1w, contrast-enhanced T1-weighted; ADC, apparent diffusion coefficient.

**Table A5 Performance indexes of radiologists and prediction models.**

| Cohort | Performance Index | Radiologist #1 | Radiologist #2 | Radiomic Signature  (Youden Cutoff) | Combined Model  (Youden Cutoff) | Clinical Model  (Youden Cutoff) |
| --- | --- | --- | --- | --- | --- | --- |
| Primary Cohort | AUC (95% CI) | 0.612 (0.539-0.684) | 0.643 (0.571-0.714) | 0.787 (0.726-0.848) | 0.826 (0.773-0.879) | 0.696 (0.619-0.773) |
| Validation Cohort | AUC (95% CI) | 0.590 (0.489-0.692) | 0.620 (0.518-0.722) | 0.783 (0.690-0.875) | 0.818 (0.731-0.905) | 0.701 (0.601-0.801) |
| Primary Cohort | Accuracy (95% CI) | 65.5% (59.9%-71.2%) | 67.4% (61.7%-73.3%) | 65.5% (59.3%-70.9%) | 71.3% (66.0%-76.6%) | 68.2% (62.4%-74.0%) |
| Validation Cohort | Accuracy (95% CI) | 66.9% (58.8%-75.1%) | 67.7% (59.8%-75.7%) | 63.8% (55.6%-71.7%) | 75.4% (68.0%-82.8%) | 68.5% (60.5%-76.3%) |
| Primary Cohort | Sensitivity (95% CI) | 53.4% (40.9%-66.6%) | 58.6% (45.8%-71.2%) | 86.2% (77.0%-95.4%) | 87.9% (79.5%-96.5%) | 58.6% (45.7%-70.9%) |
| Validation Cohort | Sensitivity (95% CI) | 44.8% (26.3%-62.9%) | 51.7% (33.7%-70.3%) | 82.8% (68.8%-96.6%) | 82.8% (68.5%-96.5%) | 51.7% (33.0%-70.9%) |
| Primary Cohort | Specificity (95% CI) | 68.9% (62.6%-75.3%) | 69.9% (63.7%-76.3%) | 59.6% (52.3%-65.9%) | 66.5% (60.2%-73.0%) | 70.9% (64.7%-77.3%) |
| Validation Cohort | Specificity (95% CI) | 73.3% (64.7%-81.9%) | 72.2% (63.5%-81.0%) | 58.4% (48.8%-67.7%) | 73.3% (64.8%-81.9%) | 73.3% (64.6%-81.6%) |
| Primary Cohort | PPV (95% CI) | 32.9% (23.8%-42.5%) | 35.8% (26.2%-45.3%) | 37.9% (29.3%-46.0%) | 42.9% (34.3%-51.5%) | 36.6% (26.5%-46.6%) |
| Validation Cohort | PPV (95% CI) | 32.5% (17.8%-46.9%) | 34.9% (20.8%-48.8%) | 36.4% (24.6%-47.6%) | 47.1% (33.2%-60.8%) | 35.7% (20.7%-50.4%) |
| Primary Cohort | NPV (95% CI) | 83.8% (78.2%-89.6%) | 85.5% (80.1%-91.0%) | 93.8% (89.5%-98.1%) | 95.1% (91.5%-98.7%) | 85.7% (80.3%-90.9%) |
| Validation Cohort | NPV (95% CI) | 82.2% (74.4%-90.1%) | 83.9% (76.3%-91.8%) | 92.2% (85.5%-98.9%) | 93.7% (88.3%-99.0%) | 84.1% (76.5%-91.9%) |

The radiologist #1 has 5 years of experience, and the radiologist #2 has 10 years of experience. Yonden cutoff values are -1.3385, 0.2117,0.1720 for radiomic signature, clinical model and combined model, respectively. AUC, area under the curve; CI, confidence interval; PPV, positive predictive value; NPV, negative predictive value.

**Table A6 Performance indexes of radiologists and prediction models in ymrT1-2 subgroup.**

| Cohort | Performance Index | Radiologist #1 | Radiologist #2 | Radiomic Signature  (Youden Cutoff) | Combined Model  (Youden Cutoff) | Clinical Model  (Youden Cutoff) |
| --- | --- | --- | --- | --- | --- | --- |
| Primary Cohort | AUC (95% CI) | 0.575 (0.419-0.699) | 0.581 (0.431-0.739) | 0.833 (0.703-0.948) | 0.874 (0.768-0.956) | 0.655 (0.515-0.795) |
| Validation Cohort | AUC (95% CI) | 0.615 (0.298-0.919) | 0.603 (0.323-0.834) | 0.915 (0.819-1) | 0.957 (0.901-1) | 0.658 (0.365-0.951) |
| Primary Cohort | Accuracy (95% CI) | 81.5% (74.2%-88.1%) | 82.6% (75.3%-90.6%) | 70.7% (60.7%-79.9%) | 68.5% (56.5%-77.4%) | 36.9% (28.2%-45.4%) |
| Validation Cohort | Accuracy (95% CI) | 85.7% (73.1%-96.1%) | 83.3% (71.9%-94.1%) | 66.7% (53.4%-82.3%) | 61.9% (47.2%-77.0%) | 21.4% (9.6%-35.5%) |
| Primary Cohort | Sensitivity (95% CI) | 25.0% (4.0%-49.0%) | 25.0% (4.0%-49.0%) | 91.7% (73.5%-100%) | 100% (100%-100%) | 91.7% (76.2%-100%) |
| Validation Cohort | Sensitivity (95% CI) | 33.3% (5.3%-94.9%) | 33.3% (5.3%-94.9%) | 100% (100%-100%) | 100% (100%-100%) | 100% (100%-100%) |
| Primary Cohort | Specificity (95% CI) | 90.0% (83.6%-97.3%) | 91.3% (85.5%-97.1%) | 67.5% (57.0%-77.8%) | 63.8% (51.9%-72.2%) | 28.7% (18.9%-37.8%) |
| Validation Cohort | Specificity (95% CI) | 89.7% (77.0%-100%) | 87.1% (77.2%-97.7%) | 64.1% (49.5%-80.8%) | 59.0% (44.1%-74.0%) | 15.4% (5.2%-26.8%) |
| Primary Cohort | PPV (95% CI) | 27.3% (5.1%-52.9%) | 30.0% (7.5%-61.7%) | 29.7% (13.8%-42.9%) | 29.3% (10.6%-46.5%) | 16.2% (8.4%-24.2%) |
| Validation Cohort | PPV (95% CI) | 20.0% (3.1%-64.9%) | 16.7% (4.3%-57.3%) | 17.6% (2.4%-36.5%) | 15.9% (0.6%-33.7%) | 8.3% (5.2%-19.8%) |
| Primary Cohort | NPV (95% CI) | 88.9% (83.1%-95.8%) | 89.0% (82.0%-96.8%) | 98.2% (94.1%-100%) | 100% (100%-100%) | 95.8% (86.7%-100%) |
| Validation Cohort | NPV (95% CI) | 94.6% (87.4%-100%) | 94.4% (85.9%-100%) | 94.6% (87.4%-100%) | 100% (100%-100%) | 100% (100%-100%) |

The radiologist #1 has 5 years of experience, and the radiologist #2 has 10 years of experience. Yonden cutoff values are -1.4208, 0.0839, 0.0897 for radiomic signature, clinical model and combined model, respectively. AUC, area under the curve; CI, confidence interval; PPV, positive predictive value; NPV, negative predictive value.

**Table A7 Performance indexes of radiologists and prediction models in ymrT3-4 subgroup.**

| Cohort | Performance Index | Radiologist #1 | Radiologist #2 | Radiomic Signature  (Youden Cutoff) | Combined Model  (Youden Cutoff) | Clinical Model  (Youden Cutoff) |
| --- | --- | --- | --- | --- | --- | --- |
| Primary Cohort | AUC (95% CI) | 0.581 (0.501-0.663) | 0.617 (0.535-0.699) | 0.756 (0.678-0.832) | 0.793 (0.723-0.860) | 0.663 (0.570-0.757) |
| Validation Cohort | AUC (95% CI) | 0.545 (0.435-0.656) | 0.584 (0.468-0.698) | 0.734 (0.616-0.850) | 0.764 (0.647-0.880) | 0.610 (0.485-0.736) |
| Primary Cohort | Accuracy (95% CI) | 56.8% (49.4%-64.3%) | 59.2% (51.6%-66.6%) | 67.5% (60.2%-74.5%) | 69.8% (62.9%-76.5%) | 75.1% (68.6%-81.7%) |
| Validation Cohort | Accuracy (95% CI) | 58.0% (47.4%-68.1%) | 60.2% (49.8%-70.4%) | 64.8% (54.8%-74.7%) | 71.6% (61.8%-81.2%) | 72.7% (63.3%-81.9%) |
| Primary Cohort | Sensitivity (95% CI) | 60.9% (47.0%-74.9%) | 67.4% (53.6%-81.1%) | 76.1% (63.9%-88.0%) | 84.8% (74.3%-95.05) | 36.9% (22.9%-50.7%) |
| Validation Cohort | Sensitivity (95% CI) | 46.2% (27.1%-65.1%) | 53.8% (33.7%-73.4%) | 73.1% (55.5%-90.4%) | 76.9% (59.7%-93.8%) | 23.1% (6.3%-39.6%) |
| Primary Cohort | Specificity (95% CI) | 55.3% (46.7%-63.9%) | 56.1% (47.2%-64.9%) | 64.2% (55.6%-72.6%) | 64.2% (55.8%-72.5%) | 89.4% (84.1%-94.9%) |
| Validation Cohort | Specificity (95% CI) | 62.9% (50.5%-74.7%) | 62.9% (50.9%-74.8%) | 61.2% (49.0%-73.5%) | 69.4% (57.7%-81.0%) | 93.5% (87.4%-99.5%) |
| Primary Cohort | PPV (95% CI) | 33.7% (23.5%-43.9%) | 36.5% (26.0%-46.8%) | 44.3% (33.1%-54.9%) | 47.0% (36.4%-57.4%) | 56.7% (38.5%-74.6%) |
| Validation Cohort | PPV (95% CI) | 34.3% (18.3%-50.0%) | 37.8% (21.8%-53.7%) | 44.2% (29.3%-59.0%) | 51.3% (35.1%-67.3%) | 60.0% (27.6%-91.5%) |
| Primary Cohort | NPV (95% CI) | 79.1% (70.7%-87.6%) | 82.1% (73.8%-90.4%) | 87.8% (81.1%-94.4%) | 91.8% (86.0%-97.6%) | 79.1% (72.5%-85.9%) |
| Validation Cohort | NPV (95% CI) | 73.6% (61.6%-85.4%) | 76.5% (64.5%-88.1%) | 84.4% (73.6%-95.2%) | 87.8% (78.2%-97.1%) | 74.4% (64.6%-83.8%) |

The radiologist #1 has 5 years of experience, and the radiologist #2 has 10 years of experience. Yonden cutoff values are -1.2177, 0.3115, 0.2249 for radiomic signature, clinical model and combined model, respectively. AUC, area under the curve; CI, confidence interval; PPV, positive predictive value; NPV, negative predictive value*.*

**Table A8 Performance indexes of prediction models based on restaging results from radiologist #1.**

|  |  | **Clinical Model** | | | **Radiomic Signature** | | | **Combined Model** | | |
| --- | --- | --- | --- | --- | --- | --- | --- | --- | --- | --- |
|  |  | **All** | **ymrT1-2** | **ymrT3-4** | **All** | **ymrT1-2** | **ymrT3-4** | **All** | **ymrT1-2** | **ymrT3-4** |
| **Primary Cohort** | **AUC** | 0.668 | 0.690 | 0.630 | 0.787 | 0.808 | 0.771 | 0.817 | 0.856 | 0.794 |
|  | **Accuracy** | 64.4% | 83.0% | 74.3% | 65.5% | 69.1% | 68.3% | 73.6% | 68.1% | 68.4% |
|  | **Sensitivity** | 58.6% | 33.3% | 32.6% | 86.2% | 86.7% | 76.7% | 86.2% | 100% | 90.7% |
|  | **Specificity** | 66.0% | 92.4% | 88.7% | 59.6% | 65.8% | 65.3% | 69.9% | 62.0% | 60.5% |
|  | **PPV** | 33.0% | 45.5% | 50.0% | 37.9% | 32.5% | 43.4% | 45.0% | 33.3% | 44.3% |
|  | **NPV** | 84.8% | 88.0% | 79.1% | 93.8% | 96.3% | 89.0% | 94.6% | 100% | 95.0% |
|  |  |  |  |  |  |  |  |  |  |  |
| **Validation Cohort** | **AUC** | 0.667 | 0.694 | 0.584 | 0.783 | 0.915 | 0.717 | 0.801 | 0.940 | 0.731 |
|  | **Accuracy** | 66.9% | 84.4% | 74.1% | 63.8% | 71.1% | 63.5% | 73.8% | 64.4% | 65.9% |
|  | **Sensitivity** | 48.3% | 33.3% | 21.7% | 82.8% | 100% | 73.9% | 72.4% | 100% | 73.9% |
|  | **Specificity** | 72.3% | 92.3% | 93.5% | 58.4% | 66.7% | 60.0% | 74.3% | 59.0% | 62.9% |
|  | **PPV** | 33.3% | 40.0% | 55.6% | 36.4% | 31.6% | 40.5% | 44.7% | 27.3% | 42.5% |
|  | **NPV** | 82.9% | 90.0% | 76.3% | 92.2% | 100% | 86.0% | 90.4% | 100% | 86.7% |

The radiologist #1 has 5 years of experience. AUC, area under the curve; CI, confidence interval; PPV, positive predictive value; NPV, negative predictive value*.*


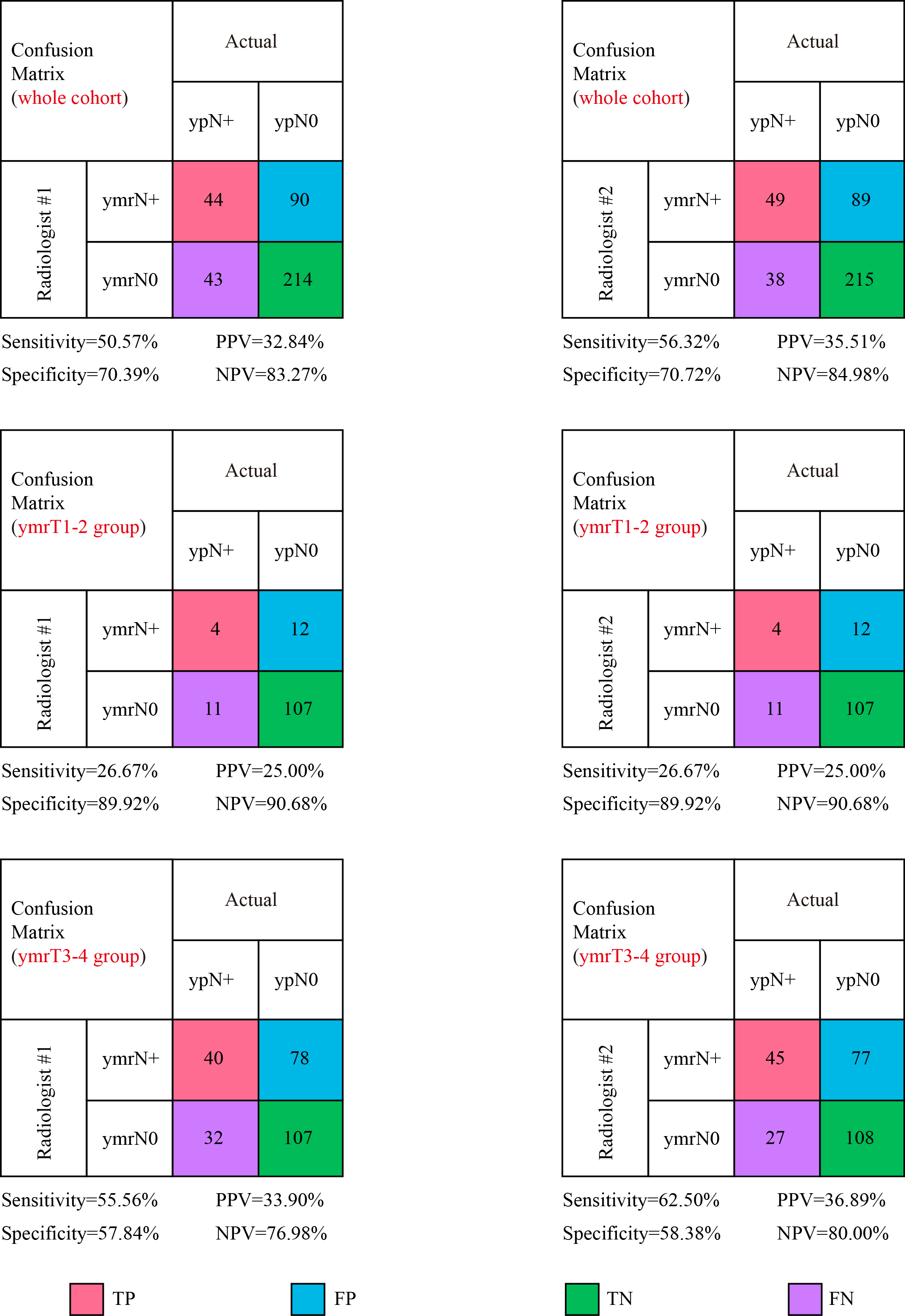


**Figure A1** Clinical MRI-based lymph node restaging (ymrN) by two radiologists compared to pathological lymph node staging (ypN) for patients who received neoadjuvant therapy. The radiologist #1 has 5 years of experience, and the radiologist #2 has 10 years of experience. TP, true positive; FP, false positive; TN, true negative; FN, false negative; PPV, positive predictive value; NPV, negative predictive value.

**
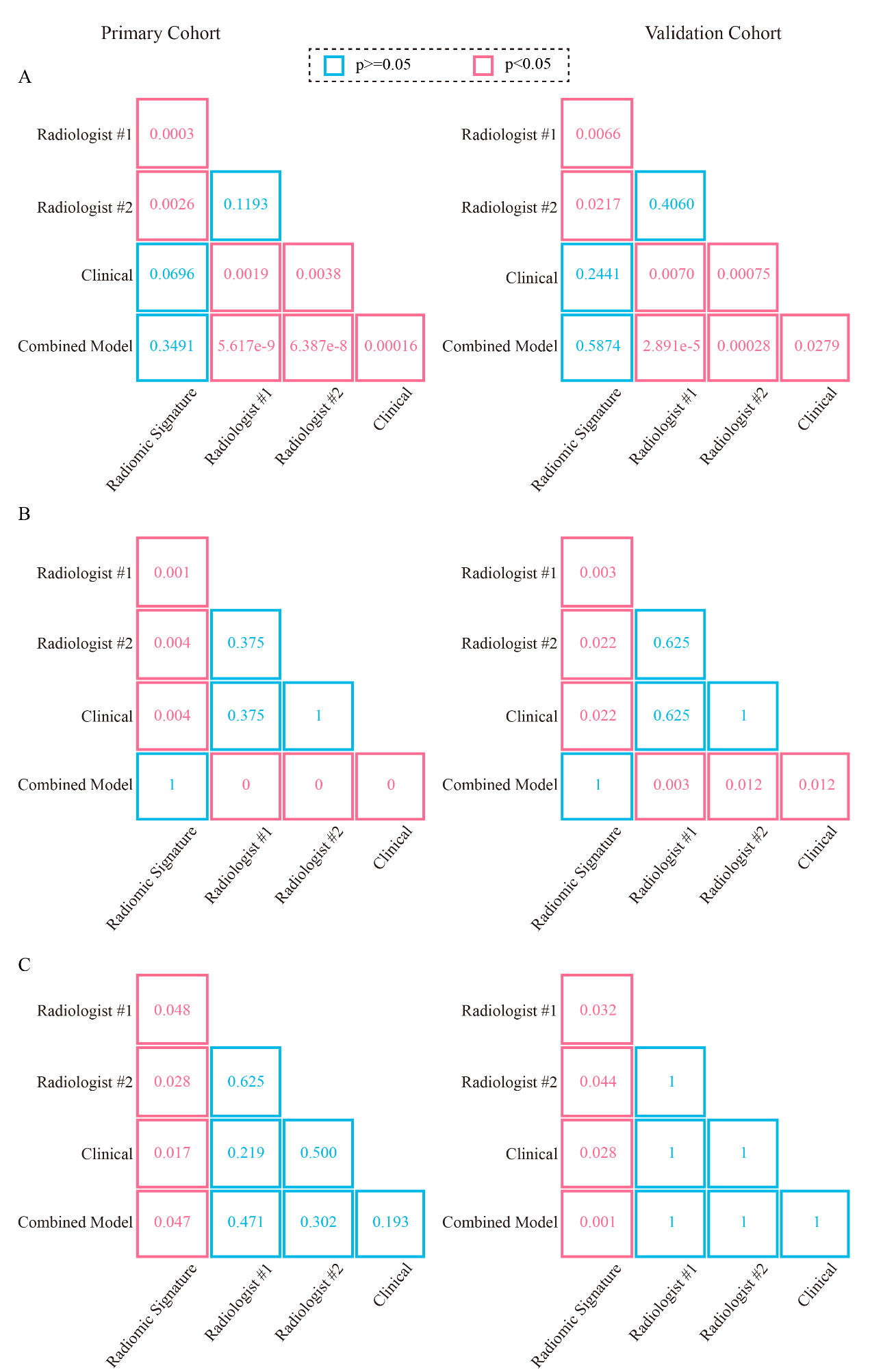
**

**Figure A2** Statistic test *p*-values in the primary cohort (left) and the validation cohort (right). (A) Delong test *p*-values for AUC; (B) Mcnemar test *p*-values for sensitivity; (C) Mcnemar test *p*-values for specificity. AUC, area under the curve.

**References**

Huang, Y.Q., Liang, C.H., He, L., Tian, J., Liang, C.S., Chen, X., et al. (2016). Development and Validation of a Radiomics Nomogram for Preoperative Prediction of Lymph Node Metastasis in Colorectal Cancer. *J Clin Oncol* 34(18)**,** 2157-2164. doi: 10.1200/jco.2015.65.9128.

Liu, Z., Zhang, X.Y., Shi, Y.J., Wang, L., Zhu, H.T., Tang, Z., et al. (2017). Radiomics Analysis for Evaluation of Pathological Complete Response to Neoadjuvant Chemoradiotherapy in Locally Advanced Rectal Cancer. *Clin Cancer Res* 23(23)**,** 7253-7262. doi: 10.1158/1078-0432.Ccr-17-1038.

Yang, L., Yang, J., Zhou, X., Huang, L., Zhao, W., Wang, T., et al. (2019). Development of a radiomics nomogram based on the 2D and 3D CT features to predict the survival of non-small cell lung cancer patients. *Eur Radiol* 29(5)**,** 2196-2206. doi: 10.1007/s00330-018-5770-y.
